# Case Report: Glomeruloid hemangioma in external jugular vein

**DOI:** 10.3389/fsurg.2025.1514289

**Published:** 2025-12-12

**Authors:** Guiping Peng, Yawen Ju, Miao Dai

**Affiliations:** 1 Department of Ultrasonic and Jiujiang CityKey Laboratory of Cell Therapy, Jiujiang NO.1 People’s Hospital, Jiujiang, Jiangxi, China; 2 Department of Geriatrics and Jiujiang CityKey Laboratory of Cell Therapy, Jiujiang NO.1 People’s Hospital, Jiujiang, Jiangxi, China; 3 Chronic Disease Management Center, Jiujiang NO.1 People’s Hospital, Jiujiang, Jiangxi, China

**Keywords:** glomerular hemangioma, external jugular vein, subcutaneous nodule, ultrasound examination, histopathological analysis

## Abstract

**Introduction:**

Glomerular hemangioma is a rare vascular tumor characterized by capillary loops that resemble glomeruli within dilated vascular spaces. While primarily reported in the skin and soft tissues, its occurrence in the external jugular vein is extremely unusual. We present a case of a 32-year-old male with a glomeruloid hemangioma located in the left external jugular vein.

**Case presentation:**

The patient presented with a small, non-tender, non-pulsatile subcutaneous nodule on the left side of the neck. There were no associated symptoms such as skin redness, swelling, or systemic signs like fever. The patient's medical history was unremarkable, with no hypertension, diabetes, or other significant conditions. Routine laboratory tests, including blood counts, electrolytes, coagulation profile, thyroid hormones, and tumor markers, were all within normal ranges. Imaging studies, including neck computed tomography, chest x-ray, and ultrasound, were performed. Ultrasound revealed a well-defined mass within the left external jugular vein with abundant blood flow and a detectable arterial blood flow spectrum. The patient underwent surgical excision of the mass, followed by histopathological examination. Ultrasound imaging showed a mass measuring 8.6 mm × 5.3 mm within the left external jugular vein, slightly hyperechoic to surrounding muscle tissue, with clear borders and regular shape. Color Doppler imaging detected abundant blood flow, indicating a vascular lesion. Histopathological analysis of the excised mass revealed capillary loops within a dilated vascular lumen, resembling glomerular structures. These findings confirmed the diagnosis of glomeruloid hemangioma.

**Conclusion:**

This case highlights a rare presentation of glomeruloid hemangioma in the external jugular vein, emphasizing the importance of considering vascular tumors in the differential diagnosis of subcutaneous neck masses. Surgical resection was successful, and histopathology provided a definitive diagnosis. This case adds to the limited reports of glomeruloid hemangiomas, particularly in atypical locations, expanding the understanding of its clinical manifestations and diagnostic approach.

## Introduction

Glomerular hemangioma is a rare benign vascular tumor that typically arises within the skin, most commonly associated with conditions such as POEMS syndrome (polyneuropathy, organomegaly, endocrinopathy, monoclonal protein, and skin changes) (1, 2). However, its occurrence in the external jugular vein is exceedingly rare, with few documented cases in the literature. Vascular tumors of the head and neck region can present diagnostic and therapeutic challenges due to their location and potential for significant vascular involvement.

The external jugular vein is a superficial vessel that drains blood from the head and neck, making it an uncommon site for primary vascular tumors. To date, hemangiomas involving the external jugular vein have rarely been reported, with most cases being of cavernous or capillary subtypes. Glomerular hemangiomas, characterized histologically by lobules of small, tightly packed capillaries resembling glomeruli, are exceptionally rare in this location. This distinct morphology is essential for differentiating glomeruloid hemangiomas from other benign vascular lesions, such as pyogenic granulomas or arteriovenous malformations.

This case report presents a unique instance of a glomeruloid hemangioma located in the external jugular vein, discussing its clinical presentation, diagnostic workup, histopathological features, and therapeutic management. By documenting this rare occurrence, we aim to enhance awareness of the diverse manifestations of glomeruloid hemangiomas and emphasize the importance of considering this entity in the differential diagnosis of vascular tumors in unusual locations. The report also highlights the relevance of histopathological examination in confirming the diagnosis, guiding appropriate treatment strategies, and preventing potential complications. This case report has been prepared in accordance with the CARE guidelines for case reports (3).

## Patient information

A 32-year-old male patient inadvertently discovered a small subcutaneous nodule on his left neck. The nodule was non-tender and non-pulsatile. There was no associated skin redness, swelling, or fever. The size of the nodule did not change with changes in the patient's position. The patient reported no other symptoms and had no history of hypertension, diabetes, or other acute or chronic diseases.

## Clinical findings

**Figure 1:**
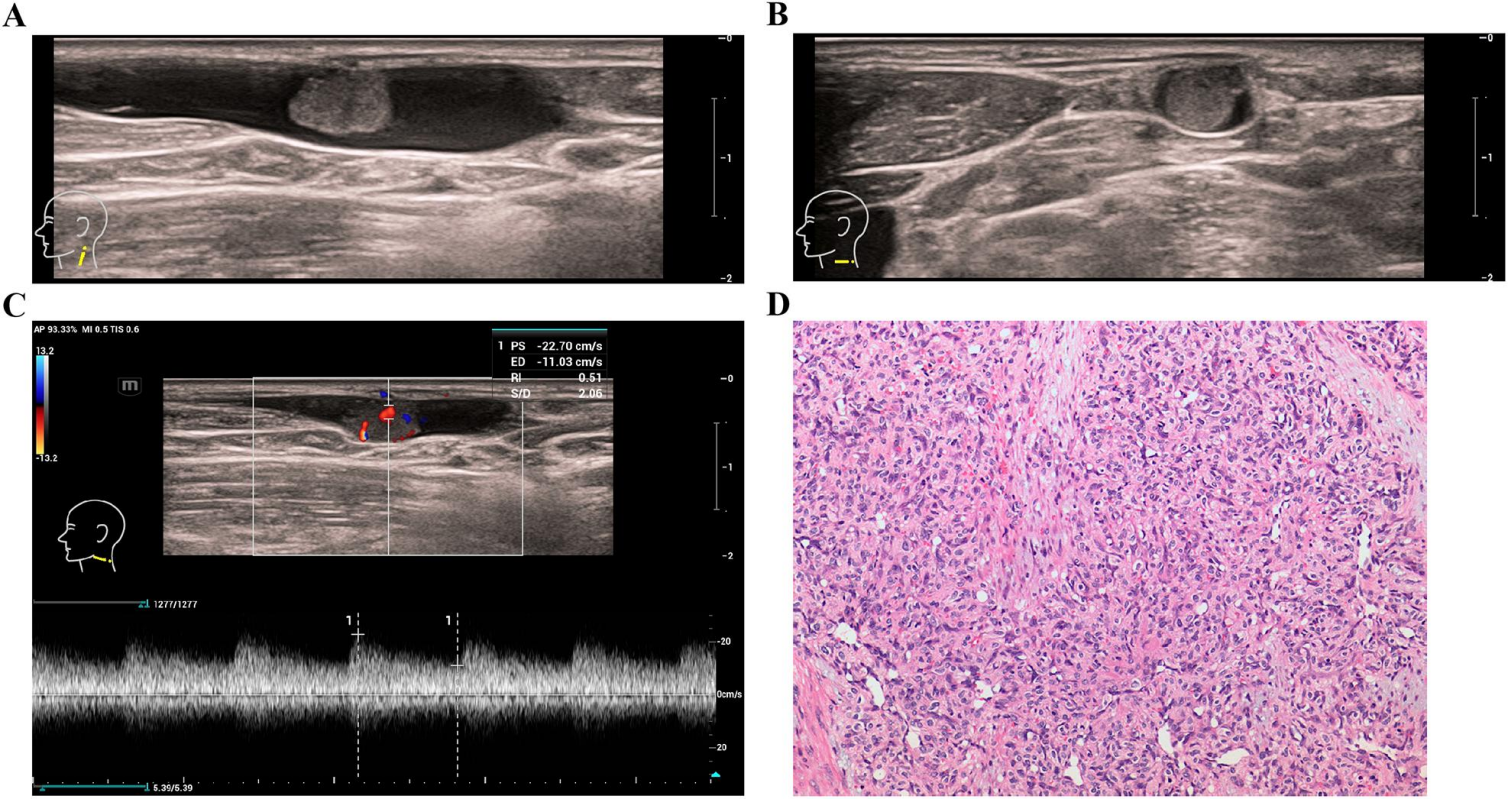
**(A)** long axis of a hypoechoic nodule within the left external jugular vein. **(B)** Short axis of a hypoechoic nodule within the left external jugular vein. **(C)** Flow spectrum of the hypoechoic nodule in the left external jugular vein. **(D)** Glomerulus-like structures within the dilated vascular lumen (hematoxylin and eosin, magnification ×200).

Upon hospitalization, routine blood tests, electrolytes, coagulation profiles, thyroid hormone levels, and tumor markers were all within normal limits. Electrocardiogram, neck CT, abdominal ultrasonography, and chest x-ray showed no abnormalities. Ultrasound examination, including 2D and color Doppler, identified a mass within the left external jugular vein, measuring 8.6 × 5.3 mm, with clear boundaries and regular shape (Figures 1A,B). The mass, slightly hyperechoic compared to the surrounding muscle tissue, exhibited abundant blood flow, with a measurable arterial blood flow spectrum (Figure 1C). The patient underwent surgical resection of the mass, and histopathological analysis revealed capillary loops resembling glomerular hyperplasia within the dilated vascular lumen, confirming a diagnosis of glomeruloid hemangioma (Figure 1D). The Timeline Summary is presented in Table 1.

**Table 1:** Timeline summary.

Time	Stage	Event
Day 1	Initial Presentation	Patient discovered a non-tender, non-pulsatile nodule on the left neck.
Day 5	Diagnostic Workup	Hospital admission; routine blood tests and imaging (ECG, CT, ultrasound) performed. Ultrasound identified an 8.6 × 5.3 mm vascular mass in the left external jugular vein.
Day 7	Treatment	Surgical resection of the mass performed.
Day 12	Confirmation	Histopathology confirmed glomeruloid hemangioma.

## Diagnostic assessment

Initial evaluations, including laboratory tests and imaging (ECG, CT, ultrasound, x-ray), were unremarkable. Ultrasound with Doppler revealed an 8.6 × 5.3 mm vascular mass in the left external jugular vein, showing abundant blood flow. Histopathology confirmed glomeruloid hemangioma via capillary loops within a dilated lumen, distinguishing it from other vascular lesions.

## Therapeutic intervention

Preoperative planning relied on high-resolution ultrasound and Color Doppler, which characterized the lesion as a well-circumscribed, hypervascular mass contained within the lumen of the external jugular vein. Although the lesion demonstrated an internal arterial flow spectrum, there was no sonographic evidence of high-flow arteriovenous shunting or direct feeding arteries from adjacent major vessels. Given its superficial location, clear intravascular confinement, and the low-pressure nature of the external jugular vein, the mass was deemed suitable for surgical excision without the need for preoperative invasive angiography. The surgical strategy involved proximal and distal control of the vein to facilitate safe *en bloc* resection. The patient underwent complete surgical excision of the mass under ultrasound guidance. The procedure was uncomplicated, with careful preservation of the external jugular vein integrity. Histopathological analysis confirmed clear margins, ensuring curative resection. No adjuvant therapy was required. This intervention highlights surgical excision as the definitive treatment for symptomatic or diagnostically uncertain glomeruloid hemangiomas.

## Follow-up and outcomes

Post-operative follow-up at 3, 6, and 12 months revealed no recurrence of the lesion. Clinical examinations and ultrasound imaging confirmed normal vascular anatomy and function of the left external jugular vein. The patient remained asymptomatic, with no residual cosmetic or functional deficits.

## Discussion

Glomerular hemangioma, as identified in this case report, is an extremely rare vascular tumor typically characterized by capillary proliferation within a dilated vascular lumen, resembling the glomeruli of the kidney. This case is notable for its unusual presentation in the external jugular vein, as glomeruloid hemangiomas have been infrequently reported and are mostly associated with systemic conditions such as POEMS syndrome (Polyneuropathy, Organomegaly, Endocrinopathy, M-protein, and Skin changes) or in cutaneous manifestations (4, 5). To our knowledge, this is one of the few documented cases of glomeruloid hemangioma occurring in a large superficial vein, emphasizing the need to consider this rare entity in the differential diagnosis of subcutaneous nodules in the neck region.

The clinical presentation in this case was subtle, with the patient noticing a small, non-tender, and non-pulsatile nodule on the left side of his neck. Unlike other vascular anomalies, the lesion did not change in size with positional changes, nor did it present with symptoms of compression or systemic signs. This presentation differs from more common vascular anomalies, such as arteriovenous malformations or cavernous venous malformations, which may exhibit pulsatility, phleboliths, or grow over time. The absence of systemic symptoms and normal laboratory findings, including tumor markers and thyroid hormones, ruled out several potential differential diagnoses, such as paragangliomas, lymphadenopathy, and metastasis.

Ultrasound imaging was crucial in the diagnostic process, as it provided information on the mass's size, location, echogenicity, and vascularity. The lesion was well-circumscribed, slightly hyperechoic, and had abundant blood flow with a detectable arterial blood flow spectrum, suggesting a vascular nature. This finding is consistent with the pathology of hemangiomas, where the presence of capillary loops within a dilated vascular structure leads to a well-defined, vascular-rich appearance on ultrasound. Given these characteristics, imaging studies can be useful in distinguishing glomeruloid hemangiomas from other soft-tissue masses, such as lipomas, cysts, or lymphatic malformations, which tend to lack prominent blood flow on Doppler examination.

Histopathological examination following surgical excision confirmed the diagnosis of glomeruloid hemangioma, revealing capillary loops resembling renal glomeruli within the vascular lumen. These capillary structures are a defining feature of this tumor and differentiate it from other benign vascular lesions. The pathophysiology behind glomeruloid hemangiomas is not entirely understood; however, it is thought to involve localized capillary hyperplasia possibly influenced by angiogenic factors. In the context of POEMS syndrome, which is often associated with glomeruloid hemangiomas, increased vascular endothelial growth factor (VEGF) levels have been implicated in capillary proliferation (2). However, this patient had no signs of POEMS syndrome, and systemic evaluation was unremarkable.

The treatment of glomeruloid hemangiomas generally involves surgical excision, especially if the lesion is symptomatic, growing, or causing cosmetic concerns, as seen in this patient. The patient expressed satisfaction with the surgical outcome, noting relief from the initial anxiety about the nodule's nature and appreciation for the thorough diagnostic process. He emphasized the importance of clear communication during treatment planning, which alleviated his concerns about potential complications. In our case, preoperative ultrasound provided adequate localization and characterization of the mass, facilitating complete resection. However, for deeper or more complex vascular lesions, intraoperative ultrasound could serve as a real-time navigation tool to enhance surgical precision, as demonstrated in neurosurgical and other specialized contexts (6). Complete surgical removal is often curative, with a low risk of recurrence, as was observed in this case. Post-operative follow-up should include regular clinical examinations to monitor for potential recurrence, particularly if the lesion is detected in unusual sites like the external jugular vein.

The unique presentation of this case raises several clinical considerations. First, it highlights the importance of maintaining a broad differential diagnosis when encountering subcutaneous nodules in the neck. While common conditions such as cysts, lipomas, and reactive lymphadenopathy may be considered first, rare entities like glomeruloid hemangiomas should also be included. This is especially relevant in cases where the mass is located within a vessel or exhibits atypical vascularity on imaging. Secondly, it underscores the role of histopathology in definitive diagnosis, given the nonspecific imaging characteristics of some vascular tumors. Surgical excision not only allows for tissue diagnosis but also serves as a definitive treatment.

## Strengths and limitations

The case utilized comprehensive imaging (ultrasound, CT) and histopathology for accurate diagnosis, ensuring definitive confirmation of glomeruloid hemangioma. Surgical excision was timely and curative, with no recurrence. The multidisciplinary approach ruled out systemic conditions like POEMS syndrome. Despite these strengths, our study has some limitations. Preoperative biopsy was not performed, which could have guided surgical planning. Long-term follow-up data are lacking to assess recurrence risk fully. The rarity of the lesion limits generalizability, and the role of non-surgical alternatives (e.g., sclerotherapy) remains unexplored.

In conclusion, this case report illustrates a rare instance of glomeruloid hemangioma located in the external jugular vein, an uncommon site for such tumors. Despite its rarity, glomeruloid hemangioma should be considered in the differential diagnosis of vascular-rich subcutaneous nodules, especially when other common conditions have been excluded. Early recognition and surgical management can lead to excellent outcomes. Further studies are needed to better understand the etiology and pathogenesis of these tumors, particularly when occurring outside their typical associations with systemic conditions like POEMS syndrome.

## Data Availability

The original contributions presented in the study are included in the article/Supplementary Material, further inquiries can be directed to the corresponding authors.
